# Medium- and Short-Term Interventions with Ma-Pi 2 Macrobiotic Diet in Type 2 Diabetic Adults of Bauta, Havana

**DOI:** 10.1155/2012/856342

**Published:** 2012-10-14

**Authors:** Carmen Porrata-Maury, Manuel Hernández-Triana, Eduardo Rodríguez-Sotero, Raúl Vilá-Dacosta-Calheiros, Héctor Hernández-Hernández, Mayelín Mirabal-Sosa, Concepción Campa-Huergo, Mario Pianesi

**Affiliations:** ^1^Clinical Assay Direction, Finlay Institute, Avenue 27, No. 19805, La Coronela, La Lisa, Havana 11600, Cuba; ^2^Latin American Nutrition Society, Iberoamerican Nutrition Foundation, and Department of Biochemistry and Physiology, Institute of Nutrition, Infanta 1158, Havana 10300, Cuba; ^3^Direction, Diabetic Care Center, Carretera Central km 29.5, Bauta 32400, Artemisa, Cuba; ^4^Lipid Metabolism Laboratory, Department of Biochemistry and Physiology, Institute of Nutrition, Infanta 1158, Havana 10300, Cuba; ^5^Presidency, Finlay Institute, Avenue 27, No. 19805, La Coronela, La Lisa, Havana 11600, Cuba; ^6^Presidency, UPM Un Punto Macrobiotico, Viale San Nicola, 62029 Tolentino, Italy

## Abstract

*Background.* In Cuba, the Ma-Pi 2 macrobiotic diet has shown positive results in 6-month assays with type 2 diabetic patients. The objective of this study was to assess the influence of this diet at short and medium terms. *Methods.* Sixty-five type 2 diabetic volunteers were included for dietary intervention, institutionally based for 21 days and followed later at home, until completing 3 months. 54 of them stayed until assay end. Before intervention, and after both assay periods, they were submitted to anthropometric records, body composition analyses and measurements of serum biochemical indicators, glycemic profile in capillary blood, blood pressure, and medication consumption; food intake was evaluated by the 3-day dietary recall. *Results.* During the intervention, the energy intake was 200 kcal higher at instance of more complex carbohydrates and dietary fiber and despite less fat and protein. Blood pressure and serum biochemical indicators decreased significantly in both periods; the safety nutritional indicators (hemoglobin, serum total proteins, and albumin) showed no variations. The global cardiovascular risk decreased and insulin consumption dropped by 46% and 64%, in both periods, respectively. *Conclusions.* The Ma-Pi 2 macrobiotic diet was a successful therapy at short term and after 3-month home-based intervention, for type 2 diabetics.

## 1. Introduction

Between 2010 and 2030, there will be a 69% increase of adults with diabetes in the developing countries [1]. The Italian International Association Un Punto Macrobiotico, UPM, founded and presided by Mario Pianesi, in collaboration with the Finlay Institute in Havana have shown positive results after 6 months of dietary institutional interventions with macrobiotic Ma-Pi 2 diet in adults affected with type 2 diabetes mellitus [2, 3]. The objective of this study was to prove those dietary effects at short term (after 21 days of controlled institutional administration) and also at medium term following 3 months during which patients remained incorporated to their daily life and were responsible for their own feeding.

## 2. Materials and Methods

The study was carried out at the Diabetic Care Center (DCC) of the municipality of Bauta, Havana, it was not a randomized double-blind trial. Instead of including a control group, which may be a limitation, a prospective cohort study was designed in 65 adult volunteers been evaluated at onset, 21 days and at 3 months at the end of the intervention. 

### 2.1. Sample Size

According to previous results [2, 3], for a power of 85% and a significance level of *α* = 0.05, a sample size of 40 individuals should be enough for detecting reductions of 26% in glycemia, 8% in total cholesterol, 12% in LDL cholesterol, and 18% in triglycerides.

### 2.2. Ethical Considerations

The study followed the recommendations of the 2000 Declaration of Helsinki [4], all participants were informed about the study procedures, patients' information was recorded in data collection charts, and the study protocol was approved by the Scientific Councils and Ethics Committees of the Finlay Institute and the Nutrition and Food Hygiene Institute (INHA, its Spanish acronym). 

### 2.3. Inclusion Criteria

Confirmed diagnosis of type 2 diabetes [5]; age between 20 and 80 years; pharmacological treatment with insulin, hypoglycemic drugs, or both; dietotherapy regimen established by the Cuban Institute of Endocrinology received; periodical DCC medical controls fulfilled and written consent of participation provided. 

### 2.4. Exclusion Criteria

Presence of concomitant factors able to modify the carbohydrates or lipid metabolism parameters (illnesses, drugs consumption), mental inability, addictions, hemoglobin <10 mg/dL, and BMI <18.5.

### 2.5. Exit Criteria

Dietary intolerance or non-acceptance of the diet, unfulfillment of the dietary medical protocol, occurence of serious events, and voluntary abandonment of the study.

### 2.6. Adverse Events

Any medical manifestation during the intervention, related or not to the diet. Anemia, low body weight, and signs or symptoms of nutritional deficiencies or excesses, were considered as events related to diet. 

### 2.7. Intervention Diet

Macrobiotic vegetarian Ma-Pi 2 diet [6, 7], designed for diseases considered with acid metabolic course. Total volume of the Ma-Pi 2 diet consisted of 40–50% whole grains (rice, millet, and barley), 40–50% vegetables (carrots, kale, cabbage, broccoli, chicory, onions, red and white radish, and parsley), and 8–10% legumes (adzuki beans, chickpeas, lentils, and black beans); all foods derived from organic cultivations with no chemical additives. Gomasio (roasted ground sesame seeds with unrefined sea salt), fermented products (miso, tamari, and umeboshi), and seaweeds (kombu, wakame, and nori) were used as complements of the diet's nutritional value. Bancha tea (theine-free green tea) was the main source of liquid.

### 2.8. Study Development

During the first 21 days, patients should assist to the DCC, where they received daily full food service daily (breakfast, lunch, dinner, and snacks), prepared by expert Ma-Pi macrobiotic cooks as well as instruction in diet preparation methods, with the purpose of guarantying their self-sufficiency at home during the next days till completing 3 months of intervention. The patients underwent medical control, daily during the first 21 days and later on every week during the first month, and every 15 days during the two following months. Information on symptoms, signs, body weight, serum glucose profile, diet compliance, and adverse events was recorded; medication doses were adjusted. 

Food consumption was evaluated by the 3-day dietary recall method (2 week days, 1 weekend day), in the three phases of the study (before the Ma-Pi diet, during the first 21 days Ma-Pi diet, and during the 3 following months). Nutrient intake was calculated with international food composition charts [8–11] and compared with dietary reference intake values (DRIs) and tolerable upper intake limits [12–14] for vitamins and minerals. Amino acid score of the protein mix was assessed using the adult amino acids requirement values proposed by Millward [15], corrected by the 80% digestibility in the Ma Pi macrobiotic diet. 

During the 3-month period, patients were supplied with whole rice, sesame as well as some small quantities of macrobiotic products as Bancha tea, seaweeds (wakame, kombu, and nori), and fermented products (miso, tamari, and umeboshi). 

At onset, 21 days, and termination (3 months), patients were submitted to the following. 

### 2.9. Anthropometric Measurements

body weight, body height, and waist and hip circumferences [16]. Body composition was also measured by bioelectrical impedance (Bioelectrical Impedance Analysis, BIO, Nutriguard-S, Serial No 103.06678, Darmstadt, Germany). Primary data were used for the calculation of the Body Mass Index (BMI = weight in kg/height in m^2^), body fat, and lean body mass. 

### 2.10. Blood Biochemical Tests

They were carried out after 12-hour fast: glucose, total cholesterol, HDL cholesterol, LDL cholesterol, triglycerides, creatinine, urea, and hepatic alanine aminotransferase (ALAT). Hemoglobin, total protein, and albumin were tested as indicators of safety. All the determinations were carried out at the INHA, following international-approved protocols. Serum glucose and lipids were determined using an Automatic Analyzer ELIMAT (SEPPIM, France), commercial reagents kits from HELFA-Diagnostics, Cuba [17], and control serums Elitrol 1-2 for quality assessment of the results. The obtained variation coefficients were 2.0% for glucose, 1.1% for total cholesterol, 2.3% for LDL cholesterol, and 2.1% for triglycerides. Hemoglobin was measured in a Coulter Electronic Cell MH, Firm ABX Micro 60, France. The rest of the biochemical measurements were carried out using an Automatic Analyzer Hitachi 902 (Roche diagnostic GmbH, Hitachi High-Technologies Corp., Japan), with commercial reagents kits from HELFA-Diagnostics, Cuba. Serum lipids were used for the estimation of the cardiovascular risk [18]. The glycemic profile in capillary blood was measured with glucometer (Roche, USA), during two whole days (three times a day: in fast, 2 hours after breakfast and two hours after lunch). Blood pressure was recorded with similar periodicity using a mercury sphygmomanometer (China). 

### 2.11. Statistical Analysis

The statistical analysis of the data was carried out using the system R, version 2.7.0 for Windows. Results were expressed in means, standard deviations, minimum and maximum values. The ANOVA test and a significance level of *P* ≤ 0.05 were used for comparisons. Graphics for data visualization were conformed using Statistica, version 7.0. 

## 3. Results

Four subjects abandoned the assay during the first 2 days because of non-acceptance of the diet. The 61 remaining patients completed the assay protocol until day 21. After this first phase, other 3 patients were excluded by apparent no fulfillment of the diet or the medical control, and other 4 requested their exclusion by different causes. Consequently, 54 (88%) of the selected patients concluded the 3-month study. The mean age of the 61 participants was 59.98 ± 10.55 years (age range 34–77 y); mean body height was 166 ± 8 cm (146–184 cm). The sample was composed by 33 women (54%) and 28 men (46%). Daily diabetic medication consumption was high at onset: 53 patients used a total of 1341 insulin units (mean consumption: 25 u/person and 0.3 u/kg Wt); 60 patients consumed 200 hypoglycemic pills (mean consumption 4 tabs/person). 

### 3.1. Nutritional Characterization of the Food Intake

A mean 200 kcal lower energy intake was recorded before intervention (20% as proteins, 30% fats, and 50% carbohydrates), distribution which met nutritional recommendations for diabetes mellitus, but composed of higher animal products (including dairy products) and fruits, scarce vegetables, and no whole cereals. The mean dietary fiber consumption was only 18 g/day. 

High whole grain cereals, vegetables, and legumes intake prevailed during intervention. At home, patients incorporated small quantities of other foods, not included in the Ma-Pi 2 diet, as yam, pumpkin, lettuce, beet, cucumber, guava, water melon, chicken meat without skin, fish, and oil. However, these small transgressions, characteristic of open studies, scarcely modified the nutritional content of the diet. The transgressions were at the minimal level while the patients were receiving their food at the DCC macrobiotic dining hall and increased at home. The individual contributions of macronutrients to the total energy intake were protein 12%, fat 16%, and carbohydrate 72%, or 12%, 18%, and 70%, in the 21 d or 3-month intervention periods, respectively, which characterizes both as low-energy-density diets. 

Mean daily energy intake increased around 200 kcal in both intervention periods. The total daily nutrients intake with the Ma-Pi diet was above two-thirds of the daily DRIs, except for vitamin B_12_, which only covered 22% of its DRI during the first intervention period and 60% in the second one, due to the addition of some animal food products. High intake levels of dietary fiber, vitamin C, folic acid, vitamin A (mainly carotenes), thiamine, pyridoxine, niacin, Mg, Mn, Zn, and adequate values of Ca intake were recorded, in spite of the absence of dairy products (Table 1). The 80% digestibility-corrected amino acid score of the ingested protein was 99 (with methionine and cystine as limiting amino acids) during the first intervention period; during the second one, this value increased to 102.

### 3.2. Effect on Anthropometric Variables

Most anthropometric records showed an apparent decrease during the 3 months, but without statistical significance (Wt, 3.7 kg; BMI, 4.9%, and waist and hip circumferences 4.2 and 3.7 cm, resp.). However, only the body fat reduction (3.1%) was close to be statistically significant (*P* = 0.059), while the lean body mass remained unchanged (Table 2). The highest observed individual value of body weight loss was 12 kg. 

### 3.3. Effect of the Diet on Biochemical Indicators

**Figure 1:**
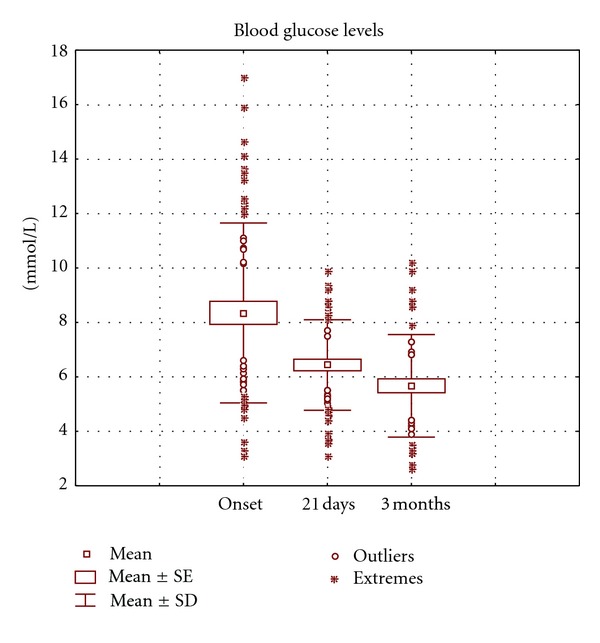
Evolution of the serum glucose levels of type 2 diabetic adults with Ma-Pi 2 diet. Note: the figure shows the mean values (squares), individual distant values (circles), and individual extreme values (asterisks) at 3 times of the assay: at onset, 21 days and 3 months after intervention with the assayed diet. The mustaches represent the standard deviation and the biggest squares the standard error of the mean. Values are expressed in mmol/L.

Serum glucose, lipids, and other indicators reflected a non-optimal metabolic control at onset. The high glycemia value at onset (8.35 mmol/L) dropped fast during the first 3 days of intervention, parallel to the insulin consumption reduction. After 21 days, the 2 mmol/L reduction (23%) was highly significant; 3 months later it was more evident (2.7 mmol/L, 32%), reaching values inside the metabolic control interval (Table 3, Figure 1).

**Figure 2:**
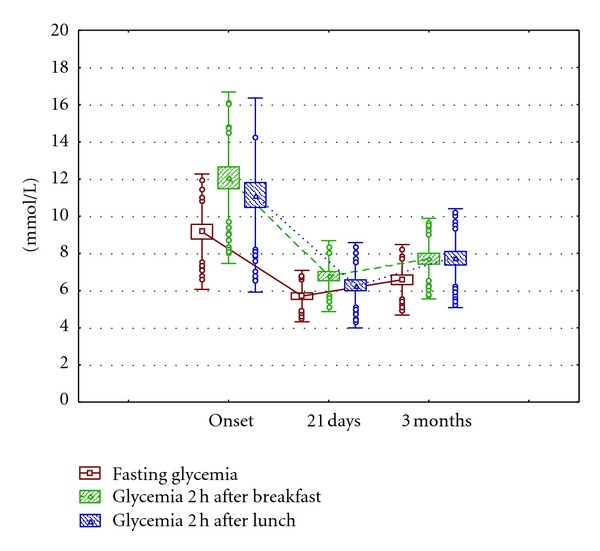
Glycemic profile evolution in type 2 diabetic adults with Ma-Pi 2 diet. Note: the figure shows the mean values (squares), individual distant values (circles), and individual extreme values (asterisks) at 3 times of the assay: at onset, 21 days and 3 months after intervention with the assayed diet. Mustaches represent the variation of the standard deviation and the biggest squares the variation of the standard error of the mean. Values are expressed in mmol/L.

The evolution of the glycemic profile evidenced also the adequate impact of the supplied diet on the control of carbohydrate metabolism (Table 4). The glycemia decreasing tendency in different moments of the day is showed in Figure 2. The data dispersion decreased during the treatment. 

**Figure 3:**
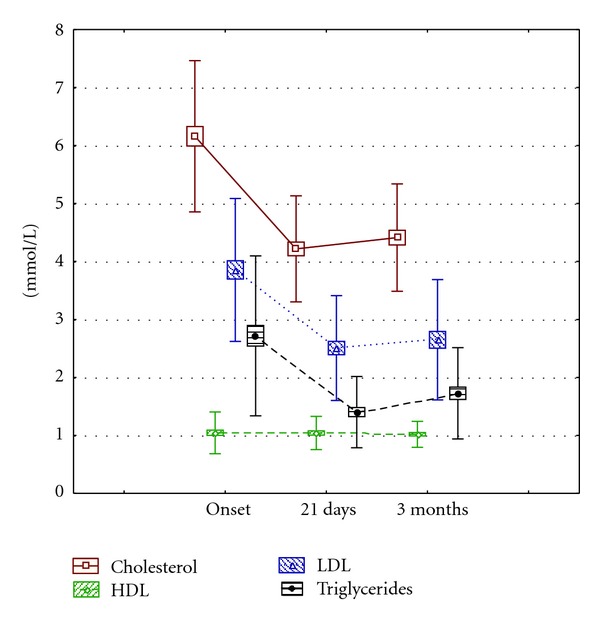
Evolution of the serum lipid indicators in type 2 diabetic adults with Ma-Pi 2 diet. Note: the squares represent the standard error of the mean and the mustaches the standard deviation. Values are expressed in mmol/L.

Serum lipids also decreased significantly after 21 days and 3 months. The HDL cholesterol values did not change significantly during the whole intervention (Table 3, Figure 3). 

At onset, the patients showed a high cardiovascular risk, in agreement with the observed serum lipid figures. The Ma-Pi 2 macrobiotic diet administration was associated to the significant cardiovascular risk decrease (Tables 3 and 5). 

From all patients included in this study, 44 (72%) had basal risk serum cholesterol levels (>5.19 mmol/L), including 31 subjects (51%) with highly risky levels (≥6.2 mmol/L). After 21 days with the diet, the numbers decreased to 8 and only one patient, respectively. This good evolution was maintained 3 months later. 

LDL cholesterol and triglycerides levels showed a similar behavior, while HDL cholesterol concentrations only evidenced a discrete and nonsignificant improvement (Table 5), which could indicate that more time is needed for an evident impact of the assayed diet. 

The lipid ratios of total cholesterol/HDL cholesterol, LDL cholesterol/HDL cholesterol, and non-HDL cholesterol/HDL cholesterol decreased after 21 days 30%, 34%, and 36%, respectively, and 3 months later they remained below the initial figures in 26%, 29%, and 32%, respectively (Table 6). The mean non-HDL cholesterol values were 5.11, 3.25, and 3.39 mmol/L, at onset, 21 days and 3 months, respectively. Other biochemical indicators also showed a significant favorable tendency at 21 d and 3 months: urea dropped by 32.8% and 21%, and the liver enzyme ALAT 18.8% and 19.5%, respectively (Table 3). 

Hemoglobin values showed a significant decrease; however, the reduction took place at expense of individual high values at onset, it can be deduced from the interval values (Table 3). 

The values of the hemoglobin, total protein, and albumin indicate that the diet can be classified as nutritionally safe, at least during the evaluated time (Table 3). 

Blood pressure decreased also significantly in a rapid way since the first 21 days (Table 2). 

### 3.4. Consumption of Medications

After 21 days, the mean insulin consumption dropped by 46% (617 units); 3 months later up to 858 units (64%) in relation to onset. The consumption of hypoglycemic pills did not show changes during the first 21 days, while 3 months later it had diminished in 10 pills (17%). 

## 4. Discussion

The fundamental therapy goals for diabetes mellitus [19] addressed to maintain low or as closer to normal as possible the values of glycemia, blood pressure, and serum lipoprotein levels, able to reduce the cardiovascular disease risk were accomplished by the results of this study. Nutritional interventions in diabetes have evidenced the capacity of diet to reduce the cardiovascular risk improving the metabolic control but only with discrete impact on drug consumption [20–23]. A recent assay in New Zealand diabetic adults [24], which reduced saturated fat and increased protein intake showed only a modest decrease of HbA1c (6%), BMI (2%), and waist circumference (2%), and medication level, without changes in glycemia, serum lipids, and blood pressure, after six months of intervention. The saturated fat intake reduction improves insulin sensitivity, independently of the energy intake [25–27]; however, a sustained high protein intake is associated with increased incidence of type 2 diabetes and diabetic nephropathy [28]. High protein intakes can increase the insulin secretion non mediated by high blood glucose concentrations [29]. A 14-year followup study in 75, 512 Hawaiian adults recently evidenced, once more, the strongly positive association of red meat intake with diabetes risk [30]. Increased plasma amino acids concentration induces directly insulin resistance in skeletal muscles and stimulates the endogenous glucose production [31]. 

The higher energy intake observed during this intervention, in comparison to previous values, would indicate that other dietary factors as energy alone should be related to the results. This Cuban study shows a diet low in fat (only 16–18% of the daily energy), low in proteins (12%), and high in whole grain cereals carbohydrates (70–72%) acting alone as powerful medication. The high fiber, Mn, Mg, and Zn intake and the reduced fat and protein content of the diet [2, 3, 7] have contributed to the observed decrease of the insulin demand. Only after 21 days, patients were able to control glycemia, serum lipids levels, and blood pressure. The fact that patients diminished further the insulin doses at 3 months indicates that they continued carrying out well enough their diet at home in spite of slightly dietary transgressions. 

Whole grain cereals dietary fiber has been associated with better peripheral insulin sensibility and also with a higher pancreas betacells secretion [32]. An intake of 50 g/day (as recorded in this study) reduces glycemia, hyperinsulinemia, hyperlipemia, and cardiovascular risk in type 2 diabetes mellitus patients [33–36]. The mean whole rice intake of the patients was 300 g a day. Whole grain rice, besides its rich content in fiber, Mg and Mn, contains 16 phyto compounds with recognized biological activity and a considerable quantity of fat soluble antioxidants like phytosterols, tocopherols and tocotrienols [37], linoleic acid (approx. 40%), oleic acid (40%), and alpha-linolenic acid, 1–3%, quantity and composition enough for guaranteeing the required *n* − 3 family polyunsaturated fatty acids supply. Tocotrienol inhibits the HMG-CoA reductase, key enzyme of the cholesterol synthesis, which awarded its potent hypolipidemic effect, besides its anticancerous and neuroprotective properties [38]. 

Discrete and nonsignificant body weight (5%) and body fat (4%) reductions were observed, parallel to the preservation of the lean body mass. In overweight and obese subjects with insulin resistance or type 2 diabetes, only a discrete body weight reduction improves insulin resistance, glycemia, and blood pressure [23, 39]. 

Hyperglycemia, hyperinsulinemia, hypertriglyceridemia, and high serum-free fatty acid levels are all characteristics of the prolonged diabetes mellitus. The maintenance of serum lipids in a normal range leads to reduced vascular complications [40] mediated by endothelial dysfunction [41]. Serum lipids and cardiovascular risk decreased significantly in this intervention. 

The hypertension levels do have predictive value in the progression prognosis of the diabetic micro- and macro-vascular complications [42]. In this study, the blood pressure control was already reached after 21 days of intervention. 

Bancha tea was the main source of liquids of the Ma-Pi diet. Green (Bancha) tea polyphenols (epigallocateqchn gallate) increase 15 times the insulin activity in vitro, protect against oxidative damage [43], and inhibit the LDL cholesterol oxidation, associated with atherosclerosis risk, heart disease, and also with the formation of reactive oxygen specimens and free radicals [44]. 

The polyphenols content of the Ma-Pi 2 diet is 2664 mg of gallic acid and the main contributors to this value are cereals 43%, vegetables 30%, leguminous 11%, and sesame seeds 8% [45] versus 1171 mg gallic acid in the Mediterranean Spanish diet, where drinks are main contributors, 50% [46]. The total antioxidant capacity of the Ma-Pi 2 diet, measured by the ABTS and FRAP methods in a parallel study, showed high values (8378 and 1571 mg ascorbic acid, resp.) where main contributing foodstuffs to this records are vegetables, 36%, and Bancha tea, 31% [45]. This value is much higher than the one reported for the Spanish Mediterranean Diet (1046 and 370 mg ascorbic acid, resp.), where drinks (wine, coffee, and tea) are the main contributors with 65% [46]. 

The possible vitamin B_12_ deficiency is an important side effect of the macrobiotic Ma-Pi 2 therapy to be considered. However, in previous Cuban studies, the serum vitamin B_12_ level was not affected after 6 months with this diet [2]. Although this vitamin is highly storage and reutilized, caution should be recommended for prolonged therapy. The 6% drop of the previous hemoglobin high values after 6-month should not have clinical translation. In a previous 6 months study in Cuban patients, anemia, microcitosis, or anisocitosis were not diagnosed and zinc protoporfirine (≥80 *μ*mol/mol hem) showed at assay end a low percentage of iron deficiency (4% versus initial 6%) [47]. Foods can no longer be evaluated only in terms of macronutrients and micronutrients content. Analyzing the content of other physiologically active components and evaluating their role in health promotion and therapeutic impact will be necessary, as it possibly happened in this intervention.

The high frequency of medical controls on these results could have an influence on the observed results; however, more attention should be perhaps addressed to the quality of nutritional advices than to the frequency of those controls. Patients included in this study were also regularly assisted at the DCC, before this intervention, for the lack of the diabetic metabolic control. 

## 5. Conclusions 

The potentiality of the Ma-Pi macrobiotic diet as therapeutic instrument for the rapid metabolic control of type 2 diabetic patients is supported by the positive effects observed at short term (21 days) on the glucose and lipid metabolism, blood pressure, body composition, and insulin consumption. The results reached at 3 months of dietary treatment, when the patient was the responsible for his own feeding, indicate the feasibility and adequacy of the offered instruction, which allowed diabetics to incorporate the learned principles to their daily feeding. The feasibility of this diet at the long term shall be assessed, especially when patients are not permanently under regular medical control. The performance of a clinical assay with a control group receiving the conventional recommended diet, which generated the metabolic results observed in these diabetic patients before intervention, is being evaluated from ethical points of view.

## Figures and Tables

**Table 1:** Average daily energy and nutrients intake during the assay periods versus Dietary Reference Intake (DRI) values and tolerable upper intake limits (ULI).

Nutrient	Average daily intake per person			DRI-ULI
	Diet at home before	Ma-Pi diet at the DCC	Ma-Pi diet at home	
Energy (kcal)	1936	2174	2144	2000
Protein (g)	98	66	64	75
Tryptophan*	13	13	13	7
Threonine*	42	35	38	29
Isoleucine*	50	41	43	34
Leucine*	80	73	75	50
Lysine*	75	42	50	35
Met + cystine*	39	34	34	31
Phen + tyrosine*	79	78	80	38
Valine*	54	50	53	26
Total fat (g)	65	38	43	44
Cholesterol (mg)	302	0	10.8	<250
Carbohydrates (g)	242	392	375	325
Fiber (g)	18	54	42	30–55
Vitamin C (mg)	94	164	211	45–2000
Folic acid (μg)	197	751	652	400–1000
Vitamin B_1_ (mg)	1.94	3.52	3.2	1.2-NA**
Vitamin B_2_ (mg)	2.08	1.30	1.1	1.3-NA**
Vitamin B_6_ (mg)	3.8	5.55	5.2	1.4-NA**
Niacin (mg)	25	25	24	16-35
Vitamin B_12_ (μg)	3.32	0.45	1.2	2.0-NA**
Vitamin E (mg)	10	10.0	11.9	9–1000
Vitamin A (μg)	2329	3266	2020	550–3000***
Potassium (mg)	3832	3646	4400	2000–3500
Manganese (mg)	2.19	16.0	14.0	2–11
Iron (mg)	11	24.0	20.7	18–53
Calcium (mg)	760	982	952	750–2500
Phosphorus (mg)	1494	1632	1804	800–4000
Zinc (mg)	10	15.4	14.3	12–40
Magnesium (mg)	325	754	720	250–350****
Sodium (mg)	3025	1724	2052	500–2300

*mg of amino acid per gram of protein, **data not available, ***preformed vitamin A only, ****tablets only.

**Table 2:** Changes of anthropometric variables and arterial blood pressure during 3-month intervention with Ma-Pi 2 macrobiotic diet in type 2 diabetic adults.

Variable	Before *n* = 61	After 21 d *n* = 61	After 3 m *n* = 54	Change After 21 d (%)	Change After 3 m (%)	*P* value
Weight (kg)	**76.2** (17.8) (47.4–133.0)	**73.5** (17.0) (44.0–128.0)	**72.5** (16.7) (42.0–121.4)	−4	−5	0.4992
BMI (kg/m^2^)	**28.8** (5.4) (21.6–48.3)	**27.7** (5.1) (20.1–46.5)	**27.4** (5.1) (19.2–44.1)	−4	−5	0.3355
Waist circumference (cm)	**95.8** (11.6) (71.0–126.0)	**93.0** (11.4) (65.0–122.0)	**91.6** (11.3) (64.0–120.0)	−3	−4	0.1278
Hip circumference (cm)	**103.5** (11.3) (86.0–136.0)	**101.4** (10.9) (84.0–134.0)	**99.7** (10.5) (84.0–134.0)	−2	−4	0.1781
Body fat (%)	**36.4** (7.0) (23.9–52.9)	**34.3** (7.0) (21.4–51.7)	**33.3** (7.5) (20.5–48.2)	−6	−9	0.0593
Lean body Mass (kg)	**45.7** (11.2) (30.5–74.0)	**45.6** (10.8) (29.8–71.3)	**45.6** (11.0) (30.4–72.7)	−0.2	−0.2	0.9993
Systolic pressure (mmHg)	**127** (19) (90–180)	**113** (12) (90–140)	**118** (7) (93–133)	−11	−7	3,888*e* ^−7^
Diastolic pressure (mmHg)	**76** (12) (57–100)	**69** (8) (50–85)	**75** (5) (63–83)	−9	−1	5,092*e* ^−5^

Values are represented as means (standard deviations), and (minimum and maximal values) *P* < 0.05 was considered significant.

**Table 3:** Changes of serum biochemical indicators during 3-month intervention with Ma-Pi 2 macrobiotic diet in type 2 diabetic adults.

Variable	Before *n* = 61	After 21 d *n* = 61	After 3 m *n* = 54	Change after 21 d (%)	Change after 3 m (%)	*P* value
Glucose (mmol/L)	**8.35** (3.3) (3.1–17.0)	**6.44** (1.7) (3.1–9.9)	**5.67** (1.9) (2.64–10.2)	−23	−32	3.533*e* ^−8^
Total cholesterol (mmol/L)	**6.2** (1.3) (3.76–9.66)	**4.3** (0.8) (3.0–5.7)	**4.4** (0.9) (2.6–6.3)	−31	−29	2.2*e* ^−16^
HDL cholesterol (mmol/L)	**1.05** (0.4) (0.4–2.1)	**1.04** (0.3) (0.6–1.8)	**1.02** (0.2) (0.7–1.5)	−5	−3	0.8889
LDL cholesterol (mmol/L)	**3.86** (1.2) (1.2–6.3)	**2.51** (0.9) (0.2–5.5)	**2.65** (1.0) (0.3–5.2)	−35	−31	1.511*e* ^−11^
Triglycerides (mmol/L)	**2.7** (1.4) (0.7–7.8)	**1.4** (0.6) (0.5–3.1)	**1.7** (0.8) (0.6–4.2)	−48	−37	1.384*e* ^−11^
Creatinine (mmol/L)	**64.1** (17.3) (33.0–117.0)	**61.2** (14.3) (38.0–106.0)	**57.7** (13.6) (24.0–90.0)	−4	−10	0.0837
Urea (mmol/L)	**5.7** (1.5) (2.6–10.7)	**3.8** (1.0) (2.0–6.8)	**4.5** (1.0) (2.0–6.7)	−33	−21	1.99*e* ^−14^
ALAT (units)	**20.3** (8.7) (8.8–47.4)	**16.5** (5.6) (7.7–29.6)	**16.4** (5.8) (7.3–30.0)	−19	−19	0.0015
Leukocytes (×10^3^/L)	**8.0** (2.1) (4.0–13.1)	**7.1** (1.8) (3.6–11.8)	**6.8** (1.6) (3.2–11.6)	−11	−15	0.0008
Hemoglobin (g/dL)	**14.1** (1.5) (10.0–18.0)	**13.4** (1.3) (10.2–16.7)	**13.3** (1.2) (10.4–15.7)	−5	−6	0.0027
Total proteins (g/L)	**76.3** (9.1) (35.9–90.8)	**74.1** (8.1) (36.5–86.4)	**76.0** (4.9) (65.1–85.8)	−3	−0.4	0.2416
Albumin (g/L)	**34.3** (4.6) (13.1–41.9)	**36.0** (4.6) (14.2–45.6)	**35.5** (5.3) (26.2–46.9)	5	3	0.1325

Values are represented as means (standard deviations), and (minimum and maximal values) *P* < 0.05 was considered significant.

**Table 4:** Glycemic profile evolution during 3-month intervention with Ma-Pi 2 diet in type 2 diabetic adults.

Variable	Time 0 *n* = 61	21 days *n* = 61	3 months *n* = 54	Change After 21 d (%)	Change after 3 m (%)	*P* value
Fasting glucose (mmol/L)	**9.2** (3.1) (3.30–17.7)	**5.7** (1.4) (3.0–8.7)	**6.6** (1.9) (3.7–11.2)	−38	−28	8.554 · *e* ^−15^
Glucose: 2 hours after breakfast (mmol/L)	**12.1** (4.6) (4.2–22.1)	**6.8** (1.91) (2.9–11.0)	**7.7** (2.17) (3.7–12.4)	−44	−36	2.200 · *e* ^−16^
Glucose: 2 hours after lunch (mmol/L)	**11.1** (5.22) (3.8–29.9)	**6.3** (2.3) (2.1–11.9)	**7.7** (2.7) (3.6–12.0)	−44	−31	2.581 · *e* ^−11^

Values are represented as means (standard deviations), and (minimum and maximal values) *P* < 0.05 was considered significant.

**Table 5:** Lipids and cardiovascular risk in adult type 2 diabetic adults with Ma-Pi 2 diet.

Variable	Cut-off points	Time 0 (%)	21 days (%)	3 months (%)
Total cholesterol (mmol/L)	Desirable <5.2	28	87	74
	Borderline 5.2–6.19	21	11	24
	High risk ≥6.20	51	2	2

LDL cholesterol (mmol/L)	Desirable <3.39	30	87	76
	Borderline 3.38–4.10	38	11	17
	High risk ≥4.11	32	3	7

HDL cholesterol (mmol/L)	Desirable >1.6	4	2	0
	Acceptable 1.0–1.6	53	55	63
	High risk ≤1.0	43	43	37

Triglycerides (mmol/L)	Desirable <1.7	33	82	78
	Borderline 1.7–2.3	35	18	20
	High 2.31–5.64	28	0	2
	High risk ≥5.65	4	0	0

Percentages of patients included in each classification according to measured serum values at onset, and after 21 d or 3 months of intervention.

**Table 6:** Serum lipid ratios related with cardiovascular risk in adult type 2 diabetic adults with Ma-Pi 2 diet.

Ratio	Before	After 21 d	After 3 m
Total cholesterol/HDL cholesterol	5.87	4.12	4.32
LDL cholesterol/HDL cholesterol	3.68	2.41	2.60
Not HDL cholesterol/HDL cholesterol	4.87	3.12	3.32
